# Exploring the Influence of Age at Menarche on Metabolic Syndrome and Its Components Across Different Women's Birth Cohorts

**DOI:** 10.1002/edm2.70015

**Published:** 2024-11-15

**Authors:** Maryam Farahmand, Maryam Mousavi, Fereidoun Azizi, Fahimeh Ramezani Tehrani

**Affiliations:** ^1^ Reproductive Endocrinology Research Center, Research Institute for Endocrine Sciences Shahid Beheshti University of Medical Sciences Tehran Iran; ^2^ Endocrine Research Center, Research Institute for Endocrine Sciences Shahid Beheshti University of Medical Sciences Tehran Iran; ^3^ Foundation for Research & Education Excellence Vestavia Hills USA

**Keywords:** generations, lifestyle factors, metabolic changes, reproductive factors

## Abstract

**Purpose:**

Metabolic syndrome (MetS) is the primary cardiovascular risk factor, making it a global issue. Our objective was to assess the association between the age at menarche (AAM) and MetS and its components in different generations of women.

**Methods:**

In this cross‐sectional study, 5500 eligible women aged ≥ 20 who participated in the Tehran lipid and glucose study in 2015–2017 were selected. Participants were divided into groups by birth cohorts (BC) (born ≤ 1959, 1960–1979, and ≥ 1980) and AAM (≤ 11, 12–15, and ≥ 16 years, early, normal, and late, respectively). The status of MetS and its components were compared amongst participants using logistic regression.

**Results:**

Normal AAM (12–15 years) was considered the reference group. The adjusted model revealed that AAM ≤ 11 is associated with a higher risk of 34% (95% confidence interval (CI): 1.04, 1.71) in MetS, and the prevalence of MetS in the early menarche group was higher in BCI, and BCII (odds ratio (OR): 1.87; 95% CI: 1.04, 3.36 and OR: 1.33; 95% CI: 1.00, 1.89, respectively). Those with late menarche demonstrated a lower risk (OR:0.72; 95% CI: 0.57, 0.91) of abdominal obesity, and early menarche showed a higher risk (OR: 1.45; CI: 1.14, 1.86). This higher risk in early menarche was observed in BCI and BCII (OR: 1.76; 95% CI: 1.16, 2.66 and OR: 1.80; 95% CI: 1.23, 2.64, respectively). However, the protective effect of late menarche was observed in BC II and BC III (OR: 0.74; 95% CI: 0.54, 1.00 and OR: 0.64; 95% CI: 0.44, 0.96, respectively).

**Conclusions:**

The influential effect of AAM on metabolic disturbances varies amongst different generations.

## Graphical abstract

The impact of age at menarche on metabolic imbalances differs across women of various generations.

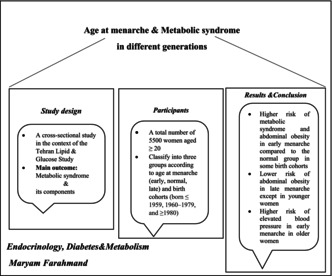

## 1 Introduction

Metabolic syndrome (MetS) is considered a global problem because it is the leading risk factor for cardiovascular disease (CVD), and its prevalence is estimated at almost 25% of the world population [1]. Furthermore, MetS is not a disease only; instead, it consists of several medical circumstances containing central obesity, Hypertension, dyslipidemia, and insulin resistance. These disorders lead to more than 60% of deaths related to noncommunicable diseases (NCDs) [1, 2]. Moreover, the prevalence of MetS is increasing globally, particularly in developing countries and amongst women. In women, reproductive factors such as menarche, and menopause can affect the MetS or its components [3, 4, 5, 6]. In Iran, the overall prevalence of MetS was reported at 29.0%, and in women and men was reported at 37.0% and 29.0%, respectively [7]. Therefore, it is necessary to know the factors affecting MetS and the incidence of its components. Today, MetS is known as a disease whose base may be in early life, so it is named “developmental origins of health and disease” [8]. So if risk factors of MetS are related to prior periods of life, like puberty fixing and controlling them can play a valuable role in prevention and on‐time interventions.

Age at menarche (AAM) is a known risk factor for MetS, as AAM can play a role in developing MetS [6, 9]. The association of AAM with metabolic disorders suggests potential mechanisms that may influence women's lifespans. However, existing studies present conflicting findings regarding this relationship. Some studies indicate an early onset of AAM is linked to MetS [6, 10]. At the same time, other research reports both early and late AAM as related to MetS [11]. In contrast, another study reported no significant association between AAM and MetS [12]. Furthermore, research results in Iran demonstrated early menarche increased the risk of some components of MetS, such as abdominal obesity, high fasting plasma glucose (FPG), and elevated blood pressure (BP) [6]. Another Asian research demonstrated an inverse association between AAM and MetS and all its components [13]. Lee et al. [14], in an umbrella review (2022), have highlighted a significant correlation between early menarche and an elevated risk of various metabolic disorders, including MetS.

The interaction between genetic and environmental factors, such as lifestyle, can affect AAM [15, 16]. On the other hand, globalisation has affected lifestyle nowadays, so all the related factors are expected to change with lifestyle changes gradually [17, 18]. Changes in the secular trend of AAM during recent decades have revealed this fact. Hence, it seems that the effect of AAM on the MetS can be different in various decades of women's lives. Although it is not clear how the AAM affects the incidence of MetS in women of varying generations, so in response to this gap, the present community‐based study was performed to evaluate the association between AAM with MetS and its components in Iranian women with a unique look at women in different BCs.

## 2 Materials and Methods

### 2.1 Study Design and Subjects

**FIGURE 1:**
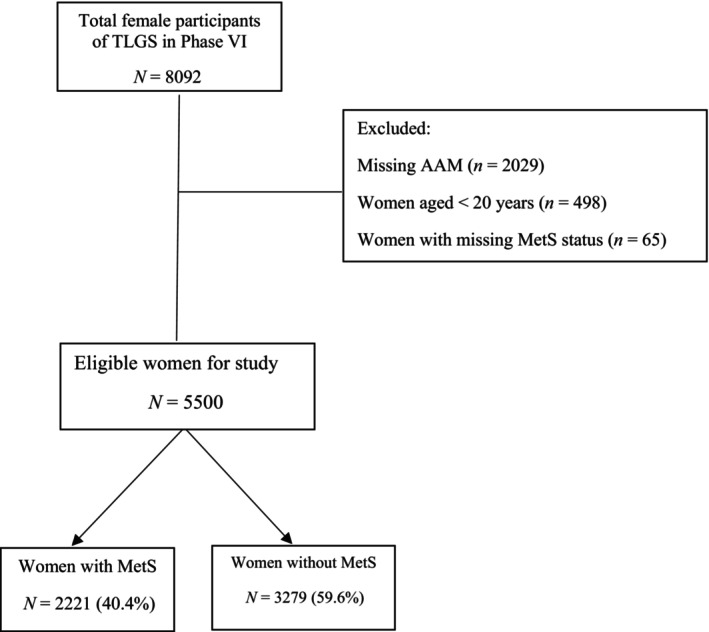
Study flowchart. MetS, metabolic syndrome; TLGS, Tehran lipid & glucose study.

The present cross‐sectional study was conducted between 2015 and 2017, from phase VI of the Tehran Lipid and Glucose Study (TLGS). TLGS was a population‐based, prospective study that began in 1998 to specify the prevalence of NCDs risk factors in an urban population in Tehran [17]. At baseline, 15,005 persons aged ≥ 3 years were picked out from a geographically restricted population. Two major components of the TLGS are a cross‐sectional study of NCDs and their risk factors and a prospective 20‐year follow‐up at 3‐year intervals. Evidence on different risk factors for NCDs, demographic variables, and reproductive histories was gathered during face‐to‐face interviews done every 3 years by trained interviewers. A comprehensive questionnaire was used for a general physical examination, height and weight measurements, and blood sample collection. Details on measurement methods have already been published [17]. The present study was the second data analysis carried out in the framework of the TLGS. According to the original study, the TLGS population is a representative sample of the urban population of Tehran, the capital of Iran. Eight thousand ninety‐two women who participated in TLGS phase VI were included (Figure 1). Amongst them, 2592 women were excluded. These excluded participants included 2029 women with missing AAM data, those aged < 20 years (498), and 65 those with missing data for MetS.

Afterward, 5500 women aged ≥ 20 years in the TLGS were divided into three groups based on their birth date using birth cohort (BC) tertiles of BC ≥ 1980, 1960–1980, and < 1960 (BCI, BCII, and BCIII, respectively) and were then divided into three groups according to AAM early, normal, and late (≤ 11, 12–15, and ≥ 16, respectively). The AAM classification was used according to other studies in TLGS and the distribution of participants' AAM [18, 19].

### 2.2 Measurements

A digital scale (Seca 707, Hanover, MD, USA) rounded to 100 g measured weight when subjects were minimally clothed. Likewise, height was measured with tape without shoes in a standing position and with normal shoulders. Waist circumference (WC) was measured by a scratched tape meter at the level of the umbilicus without any pressure on the body surface and recorded to the nearest 0.1 cm. Also, body mass index (BMI) was calculated equally as weight in kilograms (kg) divided by height squared (m^2^). Blood pressure (BP) was measured twice in the right arm after a 15‐min rest in a sitting position and was assessed according to the mean of the two measurements. The modifiable activity questionnaire evaluated physical activity. So, participants with fewer than 600 metabolic equivalent task minutes per week were classified as a low physical activity group [20]. Blood samples were gathered after a 12‐ to 14‐h overnight fasting (between 7:00 and 9:00 am). Other details for laboratory measurements were published elsewhere [17].

### 2.3 Definitions

#### 2.3.1 Exposure

The age of the first menstruation is considered the AAM and was identified by a questionnaire. The classification of AAM was conducted by calculating the 10th percentile and 90th percentile of the AAM distribution amongst TLGS participants. This method aligns with classifications utilised in other published studies [18, 19]. According to this definition, AAM was categorised as early, normal, and late, with the respective classifications being ≤ 11, 12–15, and ≥ 16 years. Birth dates were determined according to the birth certificates of the study population.

#### 2.3.2 Outcomes

MetS and its components were the outcomes of the current study. Based on the Joint Interim Statement [21], MetS was specified as the presence of any three out of the five subsequent risk factors: (1) abdominal obesity: WC ≥ 90 cm, accordingly population‐ and country‐specific cutoff for Iranians [22]; (2) high FPG ≥ 100 mg/dL or antidiabetic drug treatment, (3) High fasting triglycerides (TG) ≥ 150 mg/dL or drug treatment; (4) low fasting high‐density lipoprotein cholesterol (HDL‐C) < 50 mg/dL or drug treatment; and (5) elevated BP defined as systolic blood pressure (SBP) ≥ 130 mmHg, diastolic blood pressure (DBP) ≥ 85 mmHg or antihypertensive drug treatment.

### 2.4 Statistical

All continuous variables were checked for normality using the one‐sample Kolmogorov–Smirnov test and expressed as mean ± standard deviation if variables had a normal distribution or median with inter‐quartile range (IQ25‐75) for variables with skewed distribution. The categorical variables are expressed as numbers and percentages. The main analytical strategy to estimate the association between Mets and AAM categories (early, normal, and late) was Logistic regression analysis, which estimates odds ratios and 95% confidence intervals. The normal AAM group (12–15 years) has been considered the reference group.

This analysis was conducted in three BCs categories: BCIII (women who were born before 1960), BCII (women who were born between 1960 and 1980), and BCI (women who were born after 1980). The analyses were conducted with unadjusted and adjusted models. Adjusting variables were age, BMI (not considered for abdominal obesity and MetS), family history of diabetes, smoking status, physical activity, parity, and menopause status (just for BCII).

Statistical analysis was performed using the STATA software package (version 12; STATA Inc. College Station, TX, USA). Two‐sided *p*‐values less than 0.05 were considered statistically significant.

## 3 Results

**FIGURE 2:**
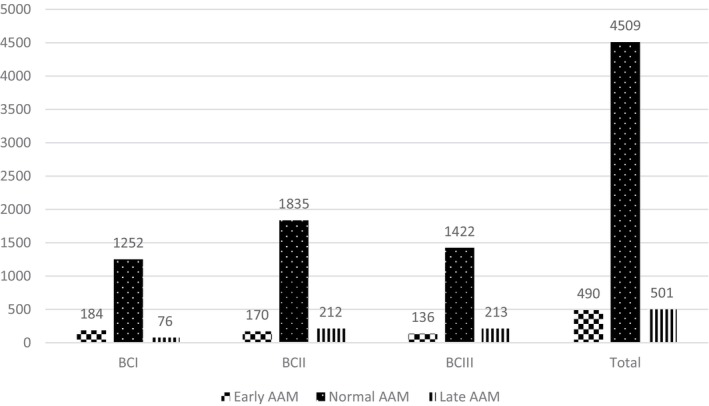
Distribution of AAM group in different birth cohorts. Early AAM is defined as AAM ≤ 11 years; normal AAM is defined as AAM between 12 and 15 years; Late AAM is defined as AAM ≥ 16 years; BCIII is defined as BC < 1960; BCII is defined as 1960 = < BC < 1980; BCI is defined as BC ≥ 1980. AAM, age at menarche; BC, birth cohort.

A total of 5500 women who were ≥ 20 years old in the 6th phase of TLGS data were included in the study, and from this, a total number of 2221 (40.4%) of women experienced MetS (Figure 1). The overall proportions of early and late AAM were 8.9% and 9.1%, respectively. The proportion of the early AAM group was higher in BCI than in BCIII (12.2% and 7.7%, respectively), while the proportion of the late AAM group was higher in BCIII than in BCI (12.0% and 5.0%, respectively). The proportion of the early and late AAM groups in BCII was 7.7% and 9.6%, respectively (Figure 2).

The characteristics of the study participants, according to the AAM grouping and MetS status, are shown in Table 1. The early AAM had the highest BMI (29.3 kg/m^2^) while owning the lowest mean current age and parity (45.0 years and 2.0, respectively). TG levels and elevated BP were higher in the late AAM group than in the early AAM group (*p* < 0.05). Most healthy women had adequate physical activity compared to those with MetS (35.3% vs. 25.7%).

**TABLE 1:** Baseline characteristics of subjects according to age at menarche and *Mets* status.

Variables	Total *N* = 5500	AAM (years)			*p*	Mets status		*p*
		≤ 11, *N* = 490 (8.9%)	12–15, *N* = 4509 (82%)	≥ 16, *N* = 501 (9.1%)		Mets, *N* = 2221 (40.4%)	Not Mets, *N* = 3279 (59.6%)	
Age (year)^a^	48.26 (15.85)	44.96 (16.64)	48.11 (15.76)	52.83 (14.85)	< 0.001	58.50 (13.06)	41.19 (13.58)	< 0.001
BMI, (kg/m^2^)^a^	28.57 (5.46)	29.37 (5.64)	28.59 (5.46)	27.62 (4.96)	< 0.001	31.63 (5.10)	26.56 (4.70)	< 0.001
Parity^b^	2.36 (2.06)	2.01 (1.94)	2.37 (2.07)	2.70 (2.11)	< 0.001	3.35 (2.20)	1.64 (1.61)	< 0.001
Physical activity (medium to high)^b^	1692 (31.4)	152 (32.1)	1380 (31.2)	160 (32.9)	0.42	555 (25.7)	1137 (35.3)	< 0.001
Family history of DM^b^	1275 (24)	101 (21.4)	1042 (24.0)	132 (27.1)	0.37	734 (33.3)	541 (17.4)	< 0.001
Smoking status (ever)^b^	202 (3.7)	28 (5.7)	155 (3.4)	19 (3.8)	0.19	78 (3.5)	124 (3.8)	< 0.001
MetS^b^	2247 (40.9)	198 (40.4)	1824 (40.5)	225 (44.9)	0.09	2221 (40.4)	3279 (59.6)	< 0.001
Abdominal obesity^b^	2978 (56.7)	266 (57.0)	2436 (56.6)	276 (57.3)	0.75	1862 (89.4)	1116 (35.2)	< 0.001
High FPG^b^	1420 (25.8)	125 (25.5)	1155 (25.6)	140 (27.9)	0.33	1203 (53.6)	217 (6.7)	< 0.001
High TG^b^	1622 (29.6)	123 (25.3)	1334 (29.7)	165 (32.9)	0.56	1280 (57.2)	342 (10.6)	< 0.001
Low HDL‐C^b^	26.27 (49.7)	229 (47.0)	2162 (48.1)	236 (47.1)	0.58	1517 (67.8)	1110 (34.2)	< 0.001
Elevated BP^b^	1453 (26.4)	133 (27.2)	1164 (25.8)	156 (31.3)	0.03	1233 (54.9)	220 (6.8)	< 0.001
Fasting								
WC (cm)^a^	93.43 (12.90)	93.72 (13.67)	93.41 (12.92)	93.38 (11.98)	0.96	101.95 (10.49)	87.84 (11.17)	< 0.001
FPG (mg/dL)^a^	98.53 (29.16)	99.33 (33.58)	98.19 (28.20)	100.78 (33.72)	0.33	112.34 (38.75)	89.01 (13.40)	< 0.001
TG (mg/dL)^c^	114.0 (80.0, 159.8)	106.0 (75.0, 151.0)	114.0 (81.0, 160.0)	120.0 (84.0, 166.0)	0.001	159.0 (118.0, 204.0)	92.0 (69.0, 124.0)	< 0.001
HDL‐C (mg/dL)^a^	50.81 (11.02)	50.94 (11.22)	50.78 (11.03)	50.97 (10.69)	0.90	46.38 (10.39)	53.87 (10.38)	< 0.001
SBP (mmHg)^a^	112.82 (18.11)	112.42 (17.83)	112.61 (17.92)	115.05 (19.82)	0.05	123.74 (18.36)	105.29 (13.51)	< 0.001
DBP (mmHg)^a^	74.36 (9.74)	74.15 (9.97)	74.28 (9.74)	75.26 (9.47)	0.19	78.22 (10.21)	71.70 (8.43)	< 0.001

*Note:* Abdominal obesity is defined as WC ≥ 90 cm; High FPG is defined as FPG ≥ 100 mg/dL or antidiabetic drug treatment; High TG is defined as TG ≥ 150 mg/dL or drug treatment; Low HDL‐C is defined as HDL‐C < 50 mg/dL in women or drug treatment; Elevated BP is defined as SBP ≥ 130 mmHg, DBP ≥ 85 mmHg or antihypertensive drug treatment. *p*‐value < 0.05 is statistically significant.

Abbreviations: AAM, age at menarche; BMI, body mass index; BP, blood pressure; DBP, diastolic blood pressure; DM, diabetes mellitus; FPG, fasting plasma glucose; HDL‐C, high‐density lipoprotein cholesterol; MetS, metabolic syndrome; SBP, systolic blood pressure; TG, triglyceride; WC, waist circumference.

^a^ Mean (SD).

^b^ Number (%).

^c^ Median (Interquartile).

Table 2 shows the ORs and 95% CIs of AAM grouping for MetS by BCs Logistic regression analysis demonstrated that the risk of MetS was 1.34 (95% CI: 1.04, 1.71) times higher in women with AAM ≤ 11 years after adjustment for potential confounders than normal AAM group. Amongst three groups of BCs, just in BCIII, the association between AAM and MetS was insignificant (OR: 1.29; 95% CI: 0.82, 2.03). At the same time, the risk of MetS amongst early AAM women in BCI (OR: 1.87; 95% CI: 1.04, 3.36) and BCII (OR: 1.33; 95% CI: 1.00, 1.89) groups was significantly higher compared to the normal AAM group.

**TABLE 2:** Odds ratios (95% confidence intervals) of AAM grouping for *MetS* by birth cohorts.

Model	Variables	Total, OR (95% CI) (*N* = 5500)	Birth cohorts		
			BC ≥ 1980 (BCI), OR (95% CI) (*N* = 1512)	1960 = < BC < 1980 (BCII), OR (95% CI) (*N* = 2217)	BC ≥ 1980 (BCI), OR (95% CI) (*N* = 1771)
Unadjusted	Early AAM, ref.: Normal	0.99 (0.83, 1.21)	1.35 (0.81, 2.23)	1.30 (0.95, 1.79)	1.29 (0.79, 1.87)
	Late AAM, ref.: Normal	1.22 (1.00, 1.44)	0.61 (0.22, 1.71)	0.97 (0.72, 1.31)	0.74 (0.54, 1.01)
Adjusted	Early AAM, ref.: Normal	1.34 (1.04, 1.71)*	1.87 (1.04, 3.36)*	1.33 (1.00, 1.89)*	1.29 (0.82, 2.03)
	Late AAM, ref.: Normal	0.86 (0.68, 1.08)	0.66 (0.20, 2.18)	0.98 (0.71, 1.36)	0.73 (0.52, 1.01)
	Age at baseline, years	1.07 (1.06, 1.08)*	1.13 (1.07, 1.19)*	1.09 (1.07, 1.12)*	1.01 (0.99, 1.02)
	Family history of diabetes	1.07 (1.02, 1.14)*	0.86 (0.68, 1.51)	1.12 (1.04, 1.20)*	1.05 (0.96, 1.16)
	Physical activity at baseline	0.79 (0.68, 0.91)*	1.17 (0.86, 1.81)	0.64 (0.52, 0.78)*	0.90 (0.69, 1.02)
	Parity, *N*	1.08 (1.03, 1.13)*	1.17 (0.93, 1.47)	1.13 (1.05, 1.23)*	1.08 (1.02, 1.15)*
	Smoking status (ever)	0.83 (0.58, 1.19)	1.38 (0.52, 3.68)	0.77 (0.45, 1.30)	0.83 (0.46, 1.48)
	Menopause status, Yes	1.64 (1.33, 2.03)*	—	1.10 (0.85, 1.43)	—

*Note:* Early AAM is defined as AAM ≤ 11 years; normal AAM is defined as AAM between 12 and 15 years; Late AAM is defined as AAM ≥ 16 years.

Abbreviations: AAM, age at menarche; BC, birth cohort; MetS, metabolic syndrome.

* *p*‐value < 0.05 is statistically significant.

Table 3 demonstrates AAM's ORs (95% confidence intervals) for components of MetS by BC groupings. Logistic regression analysis revealed that, after adjustment for potential confounders, the risk of abdominal obesity in women with AAM ≤ 11 years was 1.45 times higher (95% CI: 1.14, 1.86); this higher risk was observed in both BCI and BCII (76% and 80%, respectively), however, was insignificant in BCIII (OR: 0.87; 95% CI: 0.51, 1.48). The risk of abdominal obesity in women with AAM ≥ 16 years was 28% lower than normal AAM after adjustment for potential confounders (OR: 0.72; 95% CI: 0.57, 0.91). This protective effect was observed in both BCII (OR: 0.74; 95% CI: 0.54, 1.00) and BCIII(OR: 0.64; 95% CI: 0.44, 0.96) but not BCI (OR: 0.61; 95% CI: 0.28, 1.33). In the unadjusted model just amongst late AAM women, the risk of abdominal obesity at 39% was lower than normal AAM amongst BCIII group (95% CI: 0.41, 0.88).

**TABLE 3:** Odds ratios (95% confidence intervals) of AAM grouping for components of *MetS* by birth cohorts.

Response variables	Model	AAM group	Total, OR (95% CI) (*N* = 5500)	Birth cohorts		
				BC ≥ 1980 (BCI), OR (95% CI) (*N* = 1512)	1960 = < Birth < 1980 (BCII), OR (95% CI) (*N* = 2217)	Birth < 1960 (BCIII), OR (95% CI) (*N* = 1771)
Abdominal obesity	Unadjusted	Early	1.02 (0.84, 1.23)	1.32 (0.93, 1.87)	1.55 (1.10, 2.18)*	0.87 (0.52, 1.46)
		Late	1.03 (0.85, 1.24)	0.58 (0.30, 1.11)	0.78 (0.58, 1.04)	0.61 (0.41, 0.88)*
	Adjusted	Early	1.45 (1.14, 1.86)*	1.76 (1.16, 2.66)*	1.80 (1.23, 2.64)*	0.87 (0.51, 1.48)
		Late	0.72 (0.57, 0.91)*	0.61 (0.28, 1.33)	0.74 (0.54, 1.00)*	0.64 (0.44, 0.96)*
High FPG	Unadjusted	Early	0.99 (0.80, 1.23)	0.89 (0.44, 1.83)	1.38 (0.97, 1.95)	1.09 (0.76, 1.54)
		Late	1.12 (0.91, 1.38)	2.34 (1.12, 4.89)*	1.02 (0.73, 1.43)	0.70 (0.52, 0.94)*
	Adjusted	Early	1.08 (0.84, 1.40)	0.92 (0.37, 2.25)	1.19 (0.80, 1.77)	1.12 (0.76, 1.64)
		Late	1.04 (0.82, 1.32)	3.22 (1.30, 8.00)*	1.13 (0.78, 1.64)	0.82 (0.60, 1.13)
Elevated BP	Unadjusted	Early	1.07 (0.87, 1.32)	1.02 (0.30, 1.47)	1.46 (0.99, 2.15)	1.46 (1.00, 2.12)*
		Late	1.31 (1.07, 1.60)*	2.56 (0.74, 8.83)	0.93 (0.62, 1.38)	0.90 (0.67, 1.20)
	Adjusted	Early	1.43 (1.07, 1.90)*	0.94 (0.26, 3.39)	1.24 (0.80, 1.93)	1.74 (1.13, 2.66)*
		Late	1.07 (0.83, 1.39)	2.21 (0.47, 10.47)	1.04 (0.68, 1.60)	1.05 (0.75, 1.45)
High TG	Unadjusted	Early	0.80 (0.65, 1.99)	0.79 (0.48, 1.29)	0.80 (0.56, 1.13)	1.05 (0.73, 1.51)
		Late	1.16 (0.95, 1.42)	0.97 (0.49, 1.93)	1.04 (0.77, 1.41)	0.97 (0.72, 1.30)
	Adjusted	Early	0.80 (0.63, 1.02)	0.83 (0.46, 1.43)	0.69 (0.47, 1.01)	1.01 (0.68, 1.49)
		Late	1.19 (0.96, 1.49)	1.14 (0.46, 2.82)	1.22 (0.89, 1.68)	1.05 (0.77, 1.45)
Low HDL‐C	Unadjusted	Early	0.96 (0.79, 1.15)	0.92 (0.67, 1.26)	1.06 (0.77, 1.45)	0.90 (0.63, 1.28)
		Late	0.96 (0.80, 1.15)	0.81 (0.50, 1.29)	1.01 (0.76, 1.35)	0.94 (0.71, 1.26)
	Adjusted	Early	0.88 (0.71, 1.09)	0.79 (0.54, 1.15)	1.00 (0.71, 1.40)	0.82 (0.56, 1.21)
		Late	1.04 (0.85, 1.27)	0.86 (0.48, 1.56)	1.16 (0.86, 1.57)	0.93 (0.68, 1.28)

*Note:* Adjusted variables: age, BMI (not considered for abdominal obesity), family history of diabetes, smoking status, physical activity, parity, menopause status (just for 1960 = < BC < 1980). Early AAM is defined as AAM ≤ 11 years; normal AAM is defined as AAM between 12 and 15 years; Late AAM is defined as AAM ≥ 16 years; normal AAM is reference group; abdominal obesity is defined as WC ≥ 90 Cm; High FPG is defined as FPG ≥ 100 mg/dL or antidiabetic drug treatment; High TG is defined as TG ≥ 150 mg/dL or drug treatment; Low HDL‐C is defined as HDL‐C < 50 mg/dL in women or drug treatment; Elevated BP is defined as SBP ≥ 130 mmHg, DBP ≥ 85 mmHg or antihypertensive drug treatment.

Abbreviations: AAM, age at menarche; BC, birth cohort; BP, blood pressure; FPG, fasting plasma glucose; HDL‐C, high‐density lipoprotein cholesterol; HTN, hypertension; MetS, metabolic syndrome; TG, triglyceride.

* *p*‐value < 0.05 is statistically significant.

Logistic regression analysis revealed that the risk of high FPG did not significantly differ between the three AAM groups (*p* value > 0.05). However, in comparison between the three BC groups, just in BCI, in both unadjusted and adjusted models, the risk of high FPG in women with AAM ≥ 16 years was 2.34 times (95% CI: 1.12, 4.89), and 3.22 times (95% CI: 1.30, 8.00) higher than normal AAM group.

Table 3 shows that, after adjustment for potential confounders, the risk of elevated BP in women with AAM ≤ 11 years was 1.43 times higher (95% CI: 1.07, 1.90) than normal AAM. However, compared to the three BCs, this result was significant only in BCIII (OR: 1.74; 95% CI: 1.00, 2.12). The results for two other components of MetS, including high TG and low HDL‐c other, were not a significant association either in total participants or, according to the BCs, with any of the AAM groups (Table 3).

Furthermore, an analysis was rerun, considering AAM as a continuous variable. The results confirm that lower AAM is a risk factor for MetS overall (OR: 0.94; 95% CI: 0.90, 0.98). Moreover, the association between AAM and MetS was only significant in BCII (OR: 0.92; 95% CI: 0.87, 0.98). BCI and BCIII showed an insignificant association (OR: 0.87; 95% CI: 0.75, 1.01), (OR: 0.96; 95% CI: 0.90, 1.03), respectively.

## 4 Discussion

The present study, in conjunction with previous research [23, 24], has demonstrated a decline in the percentage of participants experiencing late AAM over generations, indicative of a broader trend of decreasing AAM over time. This reduction in AAM has been attributed to factors such as improved nutritional and health standards, increased wealth, and fattier diets [19, 25]. Our previous study amongst TLGS participants shows that the AAM decreased by 0.1 years per decade [23]. The trend towards a lower AAM in younger cohort members has been associated with various cardiometabolic risk factors [6, 26].

### 4.1 Metabolic Syndrome

The present study has identified a higher risk of metabolic syndrome (MetS) amongst participants with early AAM, which is consistent with some previous research. However, the literature on the association between AAM and MetS has reported inconsistent findings, with some studies reporting an inverse association [6, 9, 10, 14, 27, 28] and others reporting a U‐shaped association between AAM and MetS risk [11]. A comprehensive umbrella review published in 2022 includes a total of 13 reviews that encompass 283 original studies and involve over 6.8 million participants from 39 countries across five continents [14]. They found that early menarche was associated with MetS (adjusted relative risk [aRR]: 1.56, 95% CI: 1.33, 1.83); type 2 diabetes mellitus/impaired glucose tolerance (aRR: 1.30, 95% CI: 1.19, 1.42); obesity (aRR: 1.68, 95% CI: 1.53, 1.84), and hypertension (aRR: 1.24, 95% CI: 1.20, 1.29).

Interestingly, the present study found that the association between early AAM and MetS was not observed in participants belonging to the older generation. This observation agrees with other studies that have reported a changing influence of reproductive parameters on cardiometabolic diseases over time. The disappearance of the association between early AAM and MetS in the older generation may be attributed to various factors, including changes in lifestyle, nutrition, and environmental exposures, which may have modified the impact of reproductive factors on cardiometabolic risk [19, 29, 30].

### 4.2 Abdominal Obesity

The relationship between AAM and abdominal obesity is complex and may be influenced by various factors, including age, menopause status, and lifestyle. Some studies have found that late AAM may protect against abdominal obesity [14, 15], while others have not found any association [31]. The present study shows a 45% higher risk of central obesity amongst participants with early AAM, which is consistent with some previous studies [32, 33]. Furthermore, our investigation revealed that late AAM exerts a protective effect against central obesity, a significant relationship even after adjusting for potential confounders. In this regard, other studies reported that early AAM is associated with a higher risk of abdominal obesity [34, 35]. Based on different BCs, it was delineated that these associations were notably absent amongst participants from the younger generation. This shift in the impact of reproductive factors on obesity within the newer generation may be ascribed to many factors, including changes in lifestyle behaviours, dietary patterns, environmental exposures, and advancements in healthcare practices [19, 25].

Moreover, the impact of early AAM on abdominal obesity diminishes over time, with factors such as age and menopausal status assuming greater significance in the progression of abdominal obesity [30]. Our finding did not demonstrate a significant protective effect of late AAM on abdominal obesity in the younger generation(BCI), which may be because waist circumference (WC) increases with age, particularly after age 30 [36]. It suggests that the protective effect of late AAM on abdominal obesity may not be evident in younger women, even if they have a later AAM.

### 4.3 High Fasting Plasma Glucose

While several studies reported the association between AAM and MetS [6, 9, 10, 11, 27, 28], the relationship between AAM and its components, including high FPG, is less clear [37]. In the present study, the association between AAM and higher FPG, as a component of MetS, was only statistically significant amongst the young generation (BCI). We found that the risk of high FPG is increased by 3.33 and 2.34 times in those with late AAM in BCI in adjusted and unadjusted models, respectively. This observation in the younger generation may be partly explained by an overtime increase in the incidence of type 2 diabetes in younger age groups [38]. Moreover, Kjaer et al. [39] reported that women who had developed insulin‐dependent diabetes mellitus before the age of 10 years had a delayed onset of AAM by 1 year when compared to the control group. This finding suggests that late AAM may be a risk factor for the development of MetS and its components in this population. Further research is needed to confirm these findings and to explore the underlying mechanisms.

### 4.4 Elevated Blood Pressure

Our study demonstrated that after adjustment for potential confounders, early AAM is associated with a 43% increase in the risk of elevated BP as a component of MetS. Our findings showed that this added risk is only significant in the older generation (BCIII).

The association between AAM and elevated BP or Hypertension remains unclear, with inconsistent findings reported in the literature. Some studies have identified an association between early AAM and elevated BP, while others have found a link between late AAM and these conditions. Additionally, some research has reported no significant association between AAM and BP or hypertension [6, 10, 18, 40, 41]. This inconsistency in the results of studies on AAM and elevated BP can be attributed to various factors, including differences in study methods, racial and genetic variations, climate, lifestyles, and other variables that may impact the association between AAM and elevated BP [6, 18, 28, 42]. Moreover, the incidence of Hypertension increases with age, particularly in older adults [43]. Therefore, the age distribution of these cohorts can explain the lack of association between elevated BP and AAM in BCI and BCII.

### 4.5 High Triglyceride and Low High‐Density Lipoprotein Cholesterol

The present study did not find a significant association between AAM and other components of MetS, including high TG and low HDL‐C in the total population or any of the BCs. It is consistent with other studies that have reported no significant association between AAM and serum lipoproteins [44, 45]. A systematic review of the association between early puberty and lipid profile illustrated inconsistent results amongst studies, which may be due to various factors such as geographical regions, sample size, disease type, chronological age, drug use, and BMI [46]. In agreement with our findings, TG levels have been reported higher in early puberty in European populations, but no significant risk has been found in Asian populations. On the other hand, the association between HDL‐C and puberty time has been reported to be insignificant in different geographical regions [46].

In the present study, in agreement with others, we observed that early AAM is associated with a higher risk of obesity [32, 42, 47, 48]. Moreover, the present study has identified differences in reproductive characteristics amongst participants over time, including parity, which may be partially attributed to changes in family planning policies within the country and shifts in socioeconomic parameters [49].

### 4.6 Strengths and Limitations

The main strength of the current study lies in its robust methodology, utilising data derived from a large population‐based cohort study and employing rigorous assessments of MetS and its components. Furthermore, including various BCs allows for the evaluation of how the impact of AAM on MetS and its components may vary over time. Robust statistical methods were employed in this study to analyse the data and account for significant potential confounders.

One major limitation is that the population's characteristics were not assessed at the time of menarche. BMI at menarche could be a significant confounder, potentially influencing both the timing of menarche and the risk of developing MetS. Higher BMI during puberty can affect both the timing of menarche and the risk of developing metabolic disorders later in life [50]. With this data, it becomes easier to ascertain whether early AAM is directly linked to MetS or is merely a consequence of higher BMI during that developmental stage. Studies indicate that adiposity may mediate the association between early menarche and increased cardiometabolic risk factors [51, 52]. For instance, research has shown that body composition assessed in adulthood captures a significant portion of the effect of early menarche on metabolic risk factors, suggesting that earlier onset of menarche could lead to higher BMI, which in turn increases the risk for MetS [53, 54]. Future research should prioritise collecting this information to clarify these associations and better understand the underlying mechanisms involved.

We acknowledge that a significant constraint of this study is the reliance on self‐reported AAM. However, AAM is a critical developmental milestone that women typically remember well. Participants reported their AAM every 3 years, enhancing the data's consistency. According to the TLGS data, 85% of the self‐reported AAM values demonstrated adequate accuracy. For the remaining approximately 15% of participants, the earliest response or the most frequently reported AAM was utilised to determine their AAM. Given this rigorous data‐cleaning protocol, we believe that the accuracy of the AAM data is high and well‐documented.

## 5 Conclusions

In summary, the current findings underscore the significant role of AAM in MetS and some of its components. The present study found that the influential effect of AAM on MetS varies across different generations. Recognising the generational differences in how AAM influences MetS can enhance clinical practice, improve preventive care strategies, and guide future research efforts to reduce the burden of MetS in diverse populations. Further research is needed to confirm these findings across diverse populations and to explore the underlying mechanisms.

## Author Contributions

Dr. M.F. conceptualised and designed the study, designed the data‐collecting instruments, helped analyse the study results, drafted the initial manuscript, reviewed and revised the manuscript, and approved the final manuscript as submitted. Dr. M.M. helped design the study and performed all the statistical analyses. She interpreted the data, provided all the figures, and approved submitting the final manuscript. Dr. F.A. coordinated, supervised, reviewed, and approved the final manuscript as submitted. Dr. F.R.T. helped design the initial study, coordinated and supervised it, reviewed and revised the manuscript, and approved the final manuscript as submitted.

## Ethics Statement

Informed consent was obtained from all participants in accordance with the Declaration of Helsinki. The study was approved by the ethics committee of the Research Institute, Shahid Beheshti University of Medical Sciences (IR.SBMU.ENDOCRINE.REC.1401.067).

## Conflicts of Interest

The authors declare no conflicts of interest.

## Data Availability

The data that support the findings of this study are available on request from the corresponding author. The data are not publicly available due to privacy or ethical restrictions.
